# Editorial: Ubiquitin-Modifying Enzymes as Drug Targets and Biomarkers in Cancer

**DOI:** 10.3389/fonc.2021.758983

**Published:** 2021-09-15

**Authors:** Jessica L. Bell, Alena Malyukova, Jean B. Bertoldo, Matthew K. Summers, Yi Sun

**Affiliations:** ^1^Children’s Cancer Institute, School of Women’s and Children’s Health, Lowy Cancer Centre, University of New South Wales (UNSW), Sydney, NSW, Australia; ^2^Department of Cell and Molecular Biology, Karolinska Institute, Stockholm, Sweden; ^3^Institute of Molecular Medicine, Faculty of Medicine, Martin-Luther University of Halle-Wittenberg, Halle, Germany; ^4^Department of Radiation Oncology, Arthur G James Comprehensive Cancer Center and Richard L. Solove Research Institute, The Ohio State University, Columbus, OH, United States; ^5^Cancer Institute, The Second Affiliated Hospital, and Institute of Translational Medicine, Zhejiang University School of Medicine, Hangzhou, China

**Keywords:** ubiquitin, E3 ligase, cancer, proteasome, DUB

E3 ubiquitin ligases are critical to the regulatory control of a vast array of substrates and binding partners. The E3 ligases comprise the HECT, RING-finger, U-box, and PHD-finger protein families. The RING-finger family has the most members and contain ligases such as the anaphase-promoting complex (APC) and the SCF complex (Skp1-Cullin-F-box protein complex), as well as many TRIM proteins. These proteins are key regulators of cell fate in both non-malignant cells and cancer cells by adding ubiquitin to proteins and are increasingly targets for pharmaceutical modulation. They are opposed to another class of enzymes referred to as deubiquitinating enzymes (or DUBs) from protein families such as USP, UCH, OTU, JAMM, MCPIP, which also have become drug targets. The objective of this special issue was to discuss and advance the current knowledge regarding the roles and targetability of the ubiquitination machinery, in cancer. Review and research papers were accepted focusing on cancer related ubiquitin-modifying enzymes as; biomarkers, therapeutic targets, as an interacting partner of a clinically significant protein in cancer/cancer models and cancer cell phenotypes. In this Research Topic, we eventually accepted nine articles for publication (four research reviews and five research articles), which discuss the roles of ubiquitination enzymes and provide novel insights into how they impact cancer progression.

Ubiquitination plays a critical role in protein stability, MYC and p53 are probably the most reported proteins concerning their stability, being two transcription factors that are highly dynamic in expression and relatively unstable at the protein level. Although their transcriptional regulation is important, post-translational regulators of these proteins have been put forward as drug targets in cancer because direct targeting of the MYC protein or direct activation of p53 has been historically difficult. MYC, an oncoprotein, is often overexpressed and activated in tumours, and contributes to tumour aggressiveness and poor patient outcomes. Deregulation of the ubiquitination/deubiquitination balance in cancers enables MYC stabilization. In addition, SUMOylation crosstalks with the ubiquitination pathway and also controls MYC protein stability and activity. Sun et al. provided a mini-review discussing post-translation regulation of MYC which includes perspectives about MYC regulators as potential therapeutic targets in cancer. The majority of therapeutic strategies discussed by Sun et al. exploit the idea of targeting E3 ligases that promote MYC function, such as SKP2 and HUWE1 or inhibiting DUBs that antagonize MYC ubiquitination. Another promising idea presented in the review is to utilize synthetic lethality in MYC-high cancers by targeting Fbxw7 or UBR5.

p53 is a tumor suppressor protein with roles in most cancer types. It can undergo more than 300 posttranslational modifications and is principally involved in DNA damage response, apoptosis and DNA repair. Mathieu et al. present a review for this special issue on p53 and focus on the molecular mechanisms and interactions that occur between p53 and ubiquitin regulating enzymes, including irregular HECT-p53 interactions which can induce tumorigenesis. Interestingly, inhibitors developed to inhibit this interaction did not show efficacy indicating more research is needed to understand the role of HECT-p53 binding.

In this special issue there are three original research papers investigating the hepatocellular carcinoma context. One of the articles is by Li et al. and shows a new role for CISD2 in sorafenib resistance. Sorafenib is a multi-kinase inhibitor that is a standard treatment for advanced hepatocellular carcinoma, and it has previously been shown to influence protein ubiquitination. CISD2 expression is related to the progression and poor prognosis of hepatocellular carcinoma. Another aspect of hepatocellular carcinoma is initiation of the disease by viruses such as hepatitis B. Wan et al. investigated NEDD4 function in hepatitis B-induced liver cancer. They confirmed that NEDD4 induced the degradation of HBV X protein in a ubiquitin/proteasome-dependent manner *via* K48-linked ubiquitination and provided other results that together suggest NEDD4 acts as a tumor-suppressor in HBV-associated hepatocellular carcinoma. Another manuscript in the special issue investigates ubiquitin-specific proteases (USPs), which are a sub-family of DUBs. Expression of USPs are correlated with various malignancies. Ni et al. systematically investigated USPs expression in hepatocellular carcinoma. Notably, USP39 was correlated with poor prognosis in liver tumour cohorts.

DUBs are of increasing interest in cancer research and Rong et al. reviewed this class of protein, specifically the Ubiquitin C-terminal hydrolases (UCHs), a subfamily of deubiquitinating enzymes (DUBs). Their cancer context of interest is head and neck cancer, a heterogeneous disease where the long-term survival rates remain low and new drug targets are needed. They provide potential mechanisms of the UCH protein family in head and neck cancer pathogenesis and potential drug targets.

Siah2 is an E3 ubiquitin ligase that targets the androgen receptor and plays an important role in prostate cancer. Yan et al. found that the androgen receptor itself stabilizes Siah2 protein by attenuating its self-ubiquitination, likely by blocking its E3 ubiquitin ligase activity. They performed *in vivo* studies showing androgen deprivation therapy in combination with vitamin K3 delayed the development of castration-resistant prostate cancer.

Another research group made use of TCGA datasets to analyze the prognostic value of KLK8 a protein that acts as serine protease. Hua et al. showed that this protease has prognostic value in pancreatic cancer and also exerts pro-proliferation and anti-apoptotic functions in pancreatic cancer cells *via* PI3K/Akt/mTOR pathway.

Recently, a novel strategy named proteolysis-targeting chimeras (PROTACs) has been developed as a designed and targeted method of protein degradation exploiting the cancer cell’s own ubiquitin-proteasome system. This provides an additional method of drug discovery. Zhang et al. discuss and summarize the exciting advances in this field.

In summary, the collection of papers within this Research Topic offers insights into the current and emerging knowledge of the multifaceted importance of enzymes of the ubiquitin system in the context of cancer, including their biological functions and potential utility in patient care as prognostic markers, drug targets, and therapeutic tools.

## Author Contributions

All authors listed have made a substantial, direct and intellectual contribution to the work and approved it for publication.

## Funding

Funding to JLB and JBB was provided by the Wilhelm-Roux Programme (intramural funding programme of the Medical Faculty, Martin Luther University Halle-Wittenberg, Halle (Saale), Germany). Funding to MS was provided by The Ohio State University Comprehensive Cancer Center/Department of Radiation Oncology. Funding to AM was provided by Cancerföreningen i Stockholm.

## Conflict of Interest

The authors declare that the research was conducted in the absence of any commercial or financial relationships that could be construed as a potential conflict of interest.

## Publisher’s Note

All claims expressed in this article are solely those of the authors and do not necessarily represent those of their affiliated organizations, or those of the publisher, the editors and the reviewers. Any product that may be evaluated in this article, or claim that may be made by its manufacturer, is not guaranteed or endorsed by the publisher.

